# Naïve, adult, captive chimpanzees do not socially learn how to make and use sharp stone tools

**DOI:** 10.1038/s41598-023-49780-0

**Published:** 2023-12-20

**Authors:** Elisa Bandini, Claudio Tennie

**Affiliations:** 1 Department of Geosciences, Working Group Early Prehistory and Quaternary Ecology, University of Tübingen, Tübingen, Germany; 2 Department of Evolutionary Biology and Environmental Sciences, University of Zürich, Zürich, Switzerland; 3 Department of Human Evolution, Max Plank Institute for Evolutionary Anthropology, Leipzig, Germany

**Keywords:** Archaeology, Cultural evolution, Behavioural ecology

## Abstract

Although once regarded as a unique human feature, tool-use is widespread in the animal kingdom. Some of the most proficient tool-users are our closest living relatives, chimpanzees. These repertoires however consist primarily of tool *use*, rather than tool *manufacture* (for later use). Furthermore, most populations of chimpanzees use organic materials, such as sticks and leaves, rather than stones as tools. This distinction may be partly ecological, but it is also important as chimpanzees are often used as models for the evolution of human material culture, the oldest traces of which consist of manufactured sharp stone tools (so-called “flakes”). Thus, examining the conditions (if any) under which chimpanzees may develop flake manufacture and use can provide insight into the drivers of these behaviours in our own lineage. Previous studies on non-human apes’ ability to make and use flakes focused on enculturated apes, giving them full demonstrations of the behaviour immediately, without providing social information on the task in a stepwise manner. Here we tested naïve, captive chimpanzees (N = 4; three potentially enculturated and one unenculturated subject) in a social learning experimental paradigm to investigate whether enculturated and/or unenculturated chimpanzees would develop flake making and use after social information of various degrees (including a human demonstration) was provided in a scaffolded manner. Even though social learning opportunities were provided, neither the unenculturated subject nor any of the potentially enculturated subjects made or used flakes, in stark contrast to previous studies with enculturated apes. These data suggest that flake manufacture and use is outside of our tested group of captive chimpanzees’ individual and social learning repertoires. It also suggests that high levels of enculturation alongside human demonstrations (and/or training) may be required before captive chimpanzees can develop this behaviour.

## Introduction

Chimpanzees (*Pan troglodytes*) have one of the most extensive tool-use repertoires in the animal kingdom^1^. Most of the behaviours within this repertoire involve organic materials as tools (e.g., sticks and leaves), rather than inorganic materials such as stones^2^. The relatively few stone tool behaviours currently practiced by wild chimpanzees mostly involve percussive actions. For example, wild chimpanzees in Tanzania and in Senegal access encased fruit by hitting it repeatedly against stone anvils^3,4^. In Guinea, chimpanzees open hard-shelled fruit by hitting it with clubs and stone cleavers^5^^.^ Perhaps the most well-known stone tool-use behaviour in chimpanzees is nut-cracking, which in some populations involves using a stone hammer and anvil to access encased nuts (although note that some populations use wooden clubs and tree roots as anvils^6–9^). Occasionally, mishits of the stone hammer against the anvil result in small stone fragments breaking off^8,9^. In some aspects, these detached pieces bear some resemblance to hominin-produced flakes found in the archaeological record^8–12^. However, these fragments are not subsequently used by the chimpanzees as cutting tools^8,9^. This lack of interest is often explained as an absence of a need for such tools in the wild, since chimpanzees’ teeth are generally sharp enough to fulfill all their cutting needs^4–13^.

Due in part to the potential insight that can be drawn from non-human primates for our own cognitive and behavioural evolution and development^2^, researchers have investigated various species of non-human apes’ (henceforth: apes) ability to make and use flakes as cutting tools. For example, in his pioneer study on the topic, Wright^14^ provided an adult captive orangutan with the materials to make flakes (i.e., a stone hammer and suitable core) and the motivation to do so, in the form of a baited puzzle box that could only be accessed by cutting a rope keeping the door closed (see Wright^14^ for more information on the study). After human training and moulding of the behaviour, the orangutan eventually made and used flakes as cutting tools. Subsequently, Toth and colleagues^15,16^ followed a similar experimental paradigm with two captive bonobos (Kanzi and Panbanisha). After human training and demonstrations, the bonobos also started making their own flakes, using them to cut open a baited puzzle box.

Whilst these studies provide interesting data on what some species of apes are capable of after human intervention, there are limitations to the interpretation of these studies for questions focusing on the learning mechanisms driving this behaviour in apes. For example, most of these older experiments were confounded by human-introduced effects which may have impacted their results. In particular, most previous ape studies tested human-enculturated subjects (enculturated individuals are often defined as those who have been reared from a young age by humans and/or trained (intentionally or not) extensively during their lifetimes in human-oriented contexts and tasks^17^). Another major confound of these earlier studies was that the subjects were not tested first in a baseline condition, but instead immediately received human demonstrations of how to make and use sharp stone tools. Alongside the demonstrations, some subjects were trained to show the target behaviour, including in some cases by moulding of the necessary actions (i.e. the experimenter or keeper physically held the subjects arms and guided them to make the required motions^14^). Therefore, these studies do not provide data on the individual capabilities of unenculturated, untrained great apes with regards to the acquisition of flake manufacture and use abilities.

To address this latter question, Motes-Rodrigo et al.^18^ tested five captive orangutans by providing them will all the materials to make and use sharp stones in various individual and social learning conditions. The authors argue that these subjects were unenculturated and untrained as they had not been reared by humans and/or been trained in any human-oriented tasks prior to testing (see below for further discussion on enculturation). The orangutans did not make the target tools in the initial baseline condition, but in later experimental conditions three individuals engaged in lithic percussion, producing sharp stones^18^. Additionally, when provided with a (human) pre-made flake, one of the orangutans used it as a cutting tool while holding it in his mouth^18^. However, contrary to the results of the previous studies with enculturated apes, none of the orangutans in this study used stone tools that they produced themselves.

To add to the comparative database of great ape flake abilities, in a previous study^19^, we investigated whether eleven naïve, captive, chimpanzees housed across two different testing institutions (including at least eight unenculturated, untrained, subjects) would individually develop the ability to make and use flakes when provided with the necessary raw materials (“know-what”; stone cores and hammerstones) and motivation (“know-where”; a food-baited puzzle box) in a baseline condition. This experimental paradigm allowed for the investigation of flake manufacture and use, where the motivation to make the necessary tools was provided by the context of the presence of baited puzzle boxes (as in earlier studies).

Despite showing a motivation to manipulate the materials of the task and an understanding of the properties of the materials, none of the eleven chimpanzees in this previous study made or used flakes, even after they were provided with pre-made flakes (made by the experimenters out of sight of the subjects^19^). As these behaviours did not emerge in the baseline, we hypothesized that the discrepancy between these negative findings and previous positive studies (in which the full target behaviour was found) with great apes could be due to three main differences between the experimental paradigms adopted: (1) the species of apes tested (we tested chimpanzees, whilst the previous studies tested bonobos and orangutans), (2) human demonstrations and/or training in the behaviour, (3) the enculturation status of the subjects (although note that some of the subjects in our previous study may have been enculturated to some degree as well; see^19^). We assumed that it was unlikely that species had a limiting effect on the development of these behaviours, as chimpanzees currently have the most extensive tool-use repertoires of all great apes, including percussive stone tool-use behaviours^20^. Therefore, the more likely explanations included those in which enculturation and/or demonstration/training play important roles in leading to the development of these abilities in primates. Indeed, enculturation has been found to have a lasting effect on primate cognition and brain structure, allowing affected individuals to acquire abilities and behaviours not observed in their wild conspecifics, such as high levels of imitation^21–23^. For example, enculturation may have enhanced the subjects in previous studies’ imitative abilities, allowing them to copy action patterns^24^. However, enculturation can also heighten cognitive abilities that relate to increased individual learning^25^. Therefore, enculturation may have *indirectly* enabled the emergence of the target behaviour by enabling previous test subjects to make and use flakes (a human-produced zone of actual development over unenculturated apes, that would then perhaps be capable of individual knapping abilities; compare^26^). Another possibility is that enculturation equipped the subjects with the ability to copy the relevant know-how from the human demonstrations, even if these abilities were still outside of their “zones of actual development” (ZAD; see also^27–31^).

To empirically assess the relative validity of these hypotheses, two types of tests are required. The first, already conducted by^18,19^ is to provide naïve, unenculturated chimpanzees with all the raw materials and motivation to make and use flakes, but without any demonstrations or training on how to do so. As the target behaviour did not emerge in this baseline^19^, the second test involves providing different levels of social information, including know-how demonstrations (if the behaviour does not emerge after the previous types of social information are provided) to the subjects, in order to examine whether demonstration *or* enculturation is the missing factor for the emergence of these behaviours in chimpanzees. The aim of the current study was to explore this second part of the open question on how and when the target abilities emerge in chimpanzees. To do so, we provided four naïve captive chimpanzees with all the raw materials and motivation to make and use flakes (as in the baseline conducted in^19^), but we also provided social information on the task in a scaffolded manner. This process culminated in a full demonstration of the actions required for these behaviours (following the ‘step-wise’ methodology^28–31^). This approach allows for the identification of which type of social learning (if any) is required—and sufficient—before chimpanzees can develop the ability to make and use flakes.

The subjects in our study had differing rearing backgrounds, including, potentially, some level of enculturation (or deprivation; see methods section for more information on rearing histories of the subjects). It is still debated how much human interaction is required for an individual ape to become enculturated. Some argue that by virtue of living in captivity, and therefore interacting with humans on a daily basis, is enough to render an ape enculturated to some degree^32^. A more traditional view of enculturation posits that subjects must be reared by humans from a young age and/or have undergone dedicated training in human-oriented tasks before they can be deemed enculturated^17^. Thus, the extent of individual levels of enculturation is both debated, and not trivial to assess. However, at least one subject in the current study was most likely unenculturated following the traditional definition of the term (or at least less enculturated than the other chimpanzees), as he was raised by his mother and was not trained by the keepers. The other three chimpanzees in the group (with unclear backgrounds) were also tested, and data from all four subjects is discussed below (the three potentially more enculturated subjects can provide valuable information, including on the role of enculturation itself).

Overall, we tested four captive chimpanzees housed at Chimfunshi Wildlife Sanctuary across six social learning conditions. These same chimpanzees also participated in the baseline condition (lasting six hours) reported in^19^, but as they did not develop any stone tool abilities in the previous study, they were still naïve to flake manufacture and use. Furthermore, if the chimpanzees with unclear backgrounds were enculturated, this experience did not seem to have equipped them with the individual learning abilities to make and use flakes in the earlier baseline (i.e., one of the possibilities outlined above). In line with our previous findings^19^ and the results of past studies with great apes, we hypothesized that if the acquisition of flake manufacture and use abilities was due solely to the provision of social information (via humans), then the chimpanzees (including the unenculturated chimpanzee) tested would develop the full behaviours after the appropriate social learning condition was implemented. On the other hand, if enculturation is required before social information on the task can be effectively used, then only individuals with the potentially enculturated backgrounds should show the behaviour. If none of the chimpanzees develop the behaviour, even after full demonstrations are provided, then it is possible that neither enculturated nor unenculturated chimpanzees can acquire this behaviour (a species account), or that a higher level of enculturation is required than the one of the tested subjects.

## Methods

### Subjects

Four chimpanzees (mean_age_ = 29.5, range_age_: 18–46, 2F & 2 M; see Table 1) part of the ‘Escape Artists’ group housed at Chimfunshi Wildlife Sanctuary were tested. Chimfunshi is located in Zambia, in the Copperbelt region (12° 23′ S, 29° 32′ E). The chimpanzees in Escape Artists group have access to a outdoor enclosure (approx. 72 m^2^) and four indoor management areas. The outdoor area is fenced and has naturally-occurring vegetation, soil, and pebble-like stones. The chimpanzees are regularly provided with enrichment devices (e.g., cardboard tubes with food inside) and have two daily feeds, between 11.30–12.30 and 14.30–16.30. As mentioned above, the rearing histories of three of the individuals (Milla, Cleo and Chiffon) before they arrived at Chimfunshi are unclear. Before arriving at Chimfunshi, these three individuals spent some time in contact with humans, although the nature of their contact with humans is unknown. However, these three individuals spent at least a couple of years living with humans before coming to Chimfunshi. Therefore, it is possible that by virtue of spending time with humans, these chimpanzees were exposed to some level of enculturation. On the other hand, it is also possible that (some of) the subjects were kept in ‘deprived’ conditions whilst living with humans, which would have had an opposite effect to enculturation (for example, Milla was kept as a pet at a bar for some time before she was rescued, which could be regarded as deprivation). Therefore, the enculturation or deprivation status of these three individuals is difficult to confidently determine. However, the fourth chimpanzee in the group, Colin, is Cleo’s son, and was born and raised by Cleo at Chimfunshi. Colin and Cleo still live in the same social group. Therefore, we can more confidently assume that Colin was at least not as enculturated (or deprived) at the time of testing as the other three subjects. Typically, Colin would be described as unenculturated by virtue of his background.

**Table 1:** Demographic information on the subjects included in this study.

Name	Sex	Approx. DoB	Origin	Rearing
Chiffon	Male	01.01.2000	Wild	Unknown
Cleo	Female	01.01.1983	Wild	Hand
Colin	Male	05.10.1998	Captive	Mother
Milla	Female	01.01.1972	Wild	Hand

### Ethical approval

All participation was voluntary, and subjects were free to enter or leave the testing room as they wished. The chimpanzees continued with their normal feeding routine during testing. Subjects have access to water ad libitum and access to both outdoor and indoor enclosures and were never food or water deprived during testing. This project was granted ethical approval by The University of Birmingham AWERB committee (reference UOB 31213) and by the Chimfunshi Wildlife Orphanage Trust Research Board (CRAB) following SSSMZP, EAZA, BIAZA and WAZA protocols on animal research and welfare. Furthermore, this study meets all the requirements outlined by the ARRIVE guidelines.

### Materials

**Figure 1:**
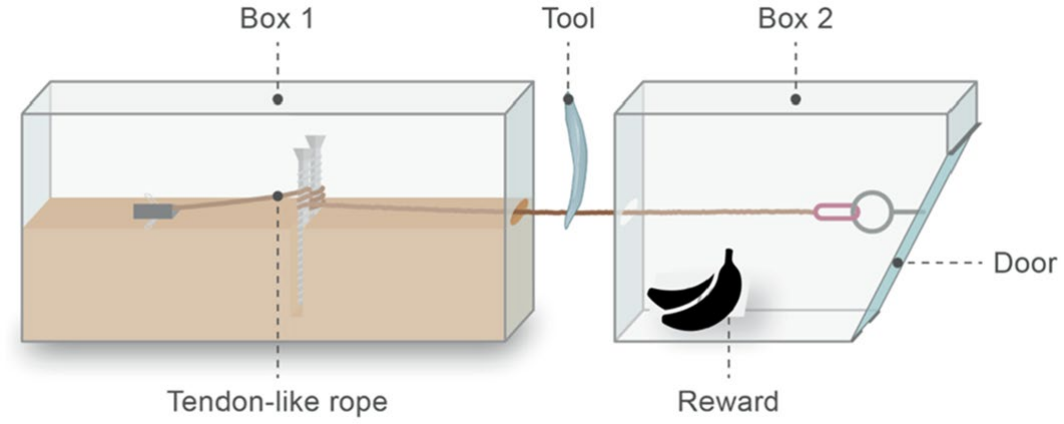
Schematic drawing of the puzzle box (courtesy of Dr. Melisa Morales; Science Graphic Design).

**Figure 2:**
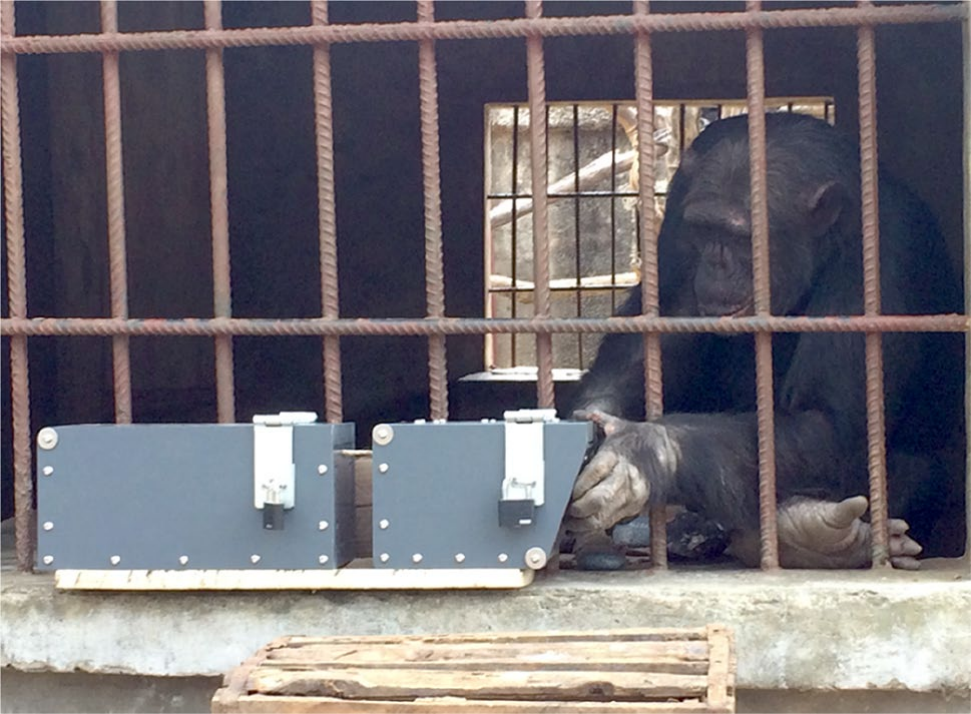
Colin manipulating the baited puzzle box in trial two of the tool exchange condition (Photograph by EB).

The subjects were tested individually and were asked to enter the testing room on a voluntary basis. Once inside the testing area, the subject had access to a baited puzzle box (which could only be opened with a cutting tool), a suitable stone core, and three hammerstones. The puzzle box was modelled on the earlier version described in^14–16^ and consisted of two boxes secured to a wooden board (see Figs. 1, 2). Box one measured (length/width/height): 36 cm × 15 cm × 17.2 cm; Box two measured (reward box): 26 cm × 17.3 cm × 17.3 cm. The reward box had a clear Plexiglas window at the top that allowed for the reward inside to be visible to the chimpanzees (measuring 5 cm × 16 cm). The door of the reward box was pulled shut by a rope that ran through the inside, exited through a hole in the opposite end where it was accessible for approx. 5 cm before running into a hole in box one. The rope was then secured in box one to a clamp that could be tightened to ensure that the rope was taut. The rope was only accessible in the area between the two boxes and had to be cut here to allow the door of box one to open. The rope was made of hemp material, approx. 2 mm thick. Collectively, the apparatus weighed approx. 21 kg (including the board). The apparatus was transported to Chimfunshi as two separate boxes, and then combined at the site by fastening the boxes to the wooden board with screws (the board measured 55.5 cm × 75 cm; distance of the puzzle box to the edge of the board: 21 cm to the sides; 4 cm to the front and back). The puzzle box was baited with peanuts, which were visible through the plexiglass window at the top of the reward box and was placed on a ledge just outside of the chimpanzees’ indoor management area (see below and^19^ for more information on the puzzle box).

**Figure 3:**
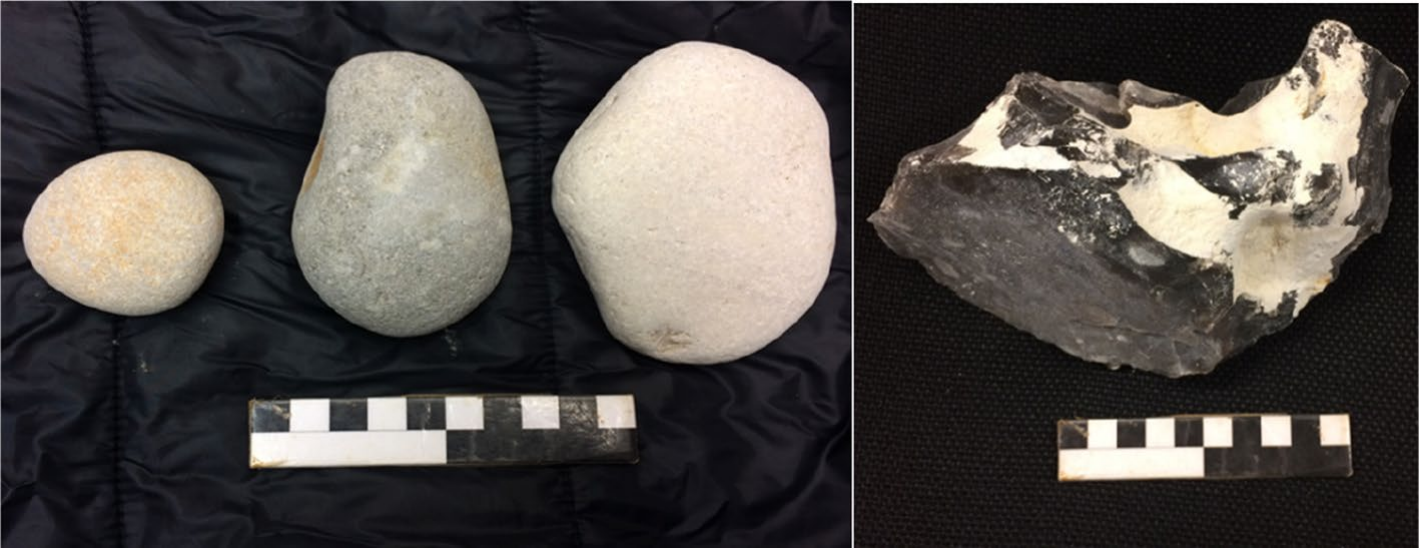
Left, photograph of three of the hammerstones provided. Right, photograph of one of the stone cores provided (Photographs by EB; scale length: 10 cm).

The stone cores were made of Norfolk flint purchased from a provider in the UK and weighed between 500 and 1500 g (see also^33^). Similarly to the previous baseline study with the chimpanzees^19^, the flint cores were first modified by an experienced knapper (W. Archer). The cores were modified to create a platform angle variability that would allow the chimpanzees to remove sharp fragments without requiring that the hammers be manipulated with precision grips (which are anatomically difficult for chimpanzees). This procedure is standard when testing non-human primates (e.g., see^15,16,19^ for further discussion on this process). The prepared cores were then packed separately and shipped to Chimfunshi. Subjects received one core per session. The hammerstones were river pebbles collected before testing at locations around the UK and shipped to Chimfunshi alongside the cores. Three ovoid hammerstones were included in each testing session, weighing between 300 and 800g^34^. Each subject received one small, medium, and large hammerstone to choose between (see Fig. 3). The three different sizes of hammerstones also allowed chimpanzees to choose the most appropriate tool for their hand size.

### Procedure

Each testing session lasted between 20 and 30 min, and each individual participated in three tests per condition. Previously, the subjects had participated in a baseline condition, in which no information other than the inclusion of the materials was provided to the subject. Following this condition, tools made out of sight of the subject were provided to the chimpanzees (see^19^ for more details on these conditions). In the current study, social information on the actions required to make and use sharp stone tools were provided in a scaffolded manner, in order to determine which type (if any) of social learning would elicit the behaviour(s). The following social learning conditions were carried-out in the listed order:

1. *Rope-cutting demonstration*: This condition provided information on the result of cutting the rope to open the door, but not on the use of flakes to cut the rope (or how to make these tools). This condition provided opportunities for stimulus and local enhancement (“know-what” and “know-where”; as the rope was being manipulated, therefore drawing the subjects’ attention to this aspect of the puzzle box) and end-state emulation (as the results of the task were being demonstrated, but not the exact actions leading to it). During this condition, the experimenter (EB) used a regular metal kitchen knife (approx. 26 cm long) to demonstrate cutting actions of the rope and subsequent door opening. EB stood near the ledge where the apparatus was placed (for the chimpanzees who were comfortable with having close contact with humans or stood one meter away with the apparatus set up on a wooden table at approx. a height of 80 cm). The subject’s attention was attracted by calling their name. Once the subject was looking towards the apparatus, EB cut the rope with the knife, opened the box, and then gave the reward to the subject to eat by placing it on the ledge outside their enclosure (see Fig. 1). The box was then rebaited and placed on the ledge, next to the hammerstones and cores for between 20 and 30 min, depending on whether the subject was still manipulating the materials after the first 20 min. These timings remained the same for all subsequent testing sessions.
2. *Loose-rope condition*: This condition was implemented to increase the subjects’ motivation to interact with the materials as they had not been successful with opening the puzzle box by that point. In this condition, the rope was left loose so that if the rope was pulled with the appropriate amount of force, even without a tool, the door to the reward box would open, allowing access to the food inside. In this condition, the chimpanzees were therefore able to access the food reward without using a tool. The hammerstones and core were still provided next to the testing apparatus, even though they were not needed. This condition again allowed for enhancement to encourage the emergence of the behaviour(s). In this step, the chimpanzees’ attention was being drawn to the rope, its location, and the fact that the rope played a role in allowing access to the bait inside the apparatus.
3. *Tool exchange condition*: This condition was provided to increase the value of flakes to examine whether the subjects would be more motivated to make their own flakes if food reward was immediate (thus, again, know-what information (via stimulus enhancement) on the tools). Three (human-)pre-made flakes measuring approx. 12 cm × 9 cm × 4 cm (made by EB out of the view of the subjects) were placed on the ledge next to the testing apparatus, hammerstones, and core before the chimpanzees entered the area. The subjects were already familiar with trading practices (*T. Calvi, personal communication*), therefore no additional training was required for the trading underlying this condition. Once the subject entered the testing room, EB pointed to one of the tools, a signal known locally to the chimpanzees in trading tasks. Once the subject handed the requested tool over, EB cut the apparatus in front of the subject using the tool and provided the reward to the subject. The apparatus was then rebaited, and this process was repeated three times, until all the tools had been exchanged. The apparatus was then placed back on the ledge where it was accessible to the subjects, alongside the hammerstones and the cores.
4. *Object movement re-enactment and results condition*: In this condition, subjects could observe how a stone dropping onto the core made a flake, but not the bodily actions of using a hammerstone to strike the core; thus this was not a full demonstration of how to make a flake. Therefore, this condition allowed for object-movement and end-state emulation, as the results of the hammer dropping were demonstrated, without bodily actions. To do so, a pulley-system was developed in which a large hammerstone (weighing approx. 1,000 g) was attached to a rope. A core was then placed on a small box approx. 5 cm from a metal mesh barrier, right under where the stone was hanging by the rope. EB hid behind the barrier, and once the chimpanzee’s attention was gained by another researcher (*L. Oña*) calling their name, the rope was released from the top of the barrier and the stone was dropped (from a height of around 50 cm) on to the core, hitting the core at an angle. This procedure was repeated until a rough usable flake was made (on average, the stone was dropped three times before a potential tool was made). The flake was then retrieved by EB and used as a tool to cut open the rope in front of the subject. The box was then opened, the reward retrieved and then passed to the subject. The puzzle box was then rebaited and placed back on the ledge, next to the hammerstones and core. The process was repeated until it was deemed likely the subject had observed one whole demonstration (assessed by observing where the subject was looking during the demonstration).
5. *Stone-drop action demonstration condition*: The aim of this condition was to further demonstrate the end product (result) of a flake by dropping a stone onto core, this time with an agent carrying out the actions of dropping a stone from above (therefore again providing opportunities for object-movement and end-state learning, but this time potentially enhanced by the presence of a human carrying out bodily actions). In this condition, EB stood in front of the enclosure with a core placed on the ground, in front of her. Once the attention of the subject had been gained by calling their name, the stone was dropped by EB from a height of approx. 1.30 m, falling on the core. This procedure was repeated until a useable flake was produced (on average the hammerstone was dropped four times before a potential tool was made). The flake was then retrieved and used as a tool to open the puzzle box and the reward was passed to the subject. The process was repeated until it was deemed likely the subject had observed one whole demonstration (assessed by observing where the subject was looking during the demonstration). The apparatus was then rebaited and placed on the ledge next to the hammerstones and core.
6. *Full demonstration condition (know-how)*: In the final demonstration condition, all the actions, goals, and results of the know-how of making and using a flake were demonstrated. This condition was similar to the one provided in previous studies on great ape stone tool manufacture and use abilities (e.g.,^14–16^). The puzzle box was baited and placed on the same wooden table as used in previous conditions in front of the enclosure alongside a core and hammerstone. The experimenter then attempted to open the door of the apparatus by pulling on it with exaggerated motions and making frustrated facial expressions (this act mimicked some of the inefficient methods used by the subjects; see results section below). EB then manipulated the rope with her fingers and moved over to the core and hammerstone. Sitting in front of the enclosure, and near to the puzzle box, the experimenter then proceeded to make a flake by striking the core at an angle (held in the left hand) with the hammerstone (held in the right hand; i.e. using the freehand knapping technique). Once a flake was produced, EB used it as a tool to cut the rope and open the door of the puzzle box. The reward was then retrieved by EB and passed to the subject. The process was repeated until it was deemed likely the subject had observed one whole demonstration (assessed by observing where the subject was looking during the demonstration). After the demonstrations were complete, the apparatus was rebaited and placed on the ledge. The hammerstones and core were placed alongside the apparatus.

### Coding of behaviours

Each condition was filmed with a Sony HDR-CX330E Handycam set up on a tripod. The video of each session was coded for any manipulation of the testing apparatus after testing. Fourteen different types of manipulation of the testing apparatus were identified. Each time the hammerstones and/or the core were manipulated was also recorded (see Table 2 for the behaviours recorded and their detailed descriptions). 25% of the videos were second-coded following the same ethogram (see Table 2, including the behavioural forms included in the “other manipulations” and “stone manipulation” categories, see results section below) by a naïve coder who was not familiar with the aim or hypothesis of the study, in order to assess the interrater reliability of the data. The second coders observations were then compared to the original ones using a Cohen’s Kappa calculation (in R version 3.4.1). Substantial agreement^35^ was found between coders, k = 0.68. All data can be accessed from the Open Science Framework platform following this link: https://osf.io/e24hk/?view_only=2d2b5ca6af2d41fd92c1da8d8e1a410c

**Table 2:** Ethogram of the manipulation types observed across conditions.

Behaviour	Description
Manipulate door	Manipulates either door of the testing apparatus with either the hands or feet, both hands and feet, or with the mouth
Manipulate locks	Manipulates the locks of the testing apparatus with either the hands or feet, both hands and feet, or with the mouth
Manipulate rope	Manipulates the rope with the hands, fingers, or mouth
Manipulate screws	Manipulates the screws of the testing apparatus with the hands or mouth, or combination of hands and mouth
Tool door	Manipulates either door of the puzzle box using a tool (e.g., one of the provided hammerstones or core)
Tool locks	Manipulates the locks of the puzzle box using a tool (e.g., one of the provided hammerstones or core)
Tool rope	Manipulates the rope using a tool (e.g., one of the provided hammerstones or a core)
Knock	Knocks on the apparatus with the knuckles
Shake	Shakes the testing apparatus violently with the hands or the feet, or a combination of hands and feet (behaviour usually results in a very loud noise)
Multiple Manip	Individual uses a series of manipulations in rapid succession
Other	Individual manipulates the apparatus in a different way as described above
Stone Manip	Individual manipulates the hammerstones or core with mouth, fingers, or hand
Pull towards	Puzzle box is pulled towards the mesh between the subject and the apparatus
Push away	Puzzle box is pushed away from the mesh between the subject and the apparatus

### Analysis

A non-parametric Mann–Kendall test (as the data was not normally distributed) was used to assess whether there was a monotonic upwards or downwards trend in the levels of manipulation of the testing apparatus over time across conditions and a Wilcoxon signed rank test (also a non-parametric test) was used to test whether there were any significant differences in time spent manipulating the rope and hammerstone and cores across the conditions. All analysis was run in R version 3.4.1.

### Experimental results

Similar to the findings of our previous study^19^, none of the chimpanzees in this study attempted to make and/or use a tool to access the puzzle box. However, in the second trial of the tool exchange condition, Cleo attempted to use a whole core, potentially to remove the rope. Cleo picked up the core and used it to *push* down on the rope, therefore not demonstrating a true ‘cutting’ action (see also^36^). Furthermore, Cleo did not seem to use any of the sharp edges of the core, but instead used the tip of the core (which was not sharp enough to cut the rope). As there was not enough space between the two boxes to allow for the use of the core (this was by experimental design, to foster flake manufacture), this attempt was unsuccessful overall. Cleo did not attempt to use the core in this manner again.

Various other manipulation types were observed (see Tables 2, 3 and 4). Overall, numerically low levels of interaction were recorded throughout the study. Out of the total 30 h of testing, subjects spent only 4:40:08 of those testing hours manipulating the apparatus (15.6%). Interaction with the testing apparatus decreased numerically across the conditions, perhaps due to the lack of success experienced by the chimpanzees. However, the difference between interaction times across conditions was not significant (Mann–Kendall; z = -0.55141, n = 1036, *p*-value = 0.5814), suggesting that the subjects maintained the same levels of motivation from the start of testing to the end, even after the loose-rope condition in which the chimpanzees were able to access the food reward easily and without the use of a tool (introduced to increase motivation). However, various types of manipulation were recorded. Subjects differed in their time spent manipulating the apparatus, and the types of preferred manipulation types (see Tables 3 and 4). Colin spent the longest time manipulating the testing apparatus (30.1% of the total testing period of this subject was spent manipulating the apparatus), followed by Milla (24.5%), Cleo (17.6%) and lastly Chiffon (8.6%).

**Table 3:** Percentage of time spent using the different manipulation types per individual.

Manip	Chiffon		Cleo		Colin		Milla	
	Time (mm:ss)	%	Time (mm:ss)	%	Time (mm:ss)	%	Time (mm:ss)	%
Knock	00:07	0	00:00	0	02:21	3	02:01	2
Manipulate door	13:20	47	17:37	40	27:07	31	36:38	45
Manipulate lock	01:38	6	05:18	12	02:15	3	06:33	8
Manipulate rope	04:18	15	05:52	13	00:47	1	17:18	21
Manipulate screws	00:00	0	01:50	4	00:19	0	02:17	3
Multiple manipulation	01:13	4	03:05	7	01:08	1	03:07	4
Other	00:00	0	02:09	5	00:20	0	04:42	6
Pull towards	00:00	0	01:34	4	01:50	2	00:13	0
Push away	01:26	5	01:53	4	00:00	0	00:00	0
Shake	00:00	0	00:12	0	39:44	46	00:00	0
Stone manipulation	04:43	17	02:47	6	08:04	9	01:49	2
Tool door	00:39	2	00:05	0	02:29	3	05:22	7
Tool lock	00:00	0	00:07	0	00:30	1	01:00	1
Tool rope	00:57	3	01:16	3	00:00	0	00:16	0

**Table 4:** Total percentage of time spent practicing “other” manipulations.

Manipulation	Time (mm:ss)	%
Change orientation	01:37	13
Manipulate wire	08:39	68
Manipulate wood	01:07	9
Peer into window	01:20	10

## Discussion

Despite various social learning conditions, culminating in a full human demonstration of flake manufacture and use through various techniques (e.g., stone throwing/dropping and freehand percussion), none of the four chimpanzees in our sample made or used a flake in any of the conditions. This outcome is in contrast to the positive results of previous studies who demonstrated (and sometimes trained and moulded) the necessary abilities to enculturated apes^14–16^, and to the findings of a recent study in which unenculturated orangutans showed both of the target skills—but not in combination—during play/exploration, in the absence of previous know-how demonstrations^18^.

Although we did not know the exact level of enculturation of all subjects, it is likely that one individual (Colin) was not enculturated (or, following the stricter definitions of enculturation, was minimally enculturated by virtue of being in captivity). Therefore, we paid particular attention to this individual. However, the fact that all four chimpanzees failed to show the target behaviours after demonstrations suggests that higher levels of enculturation than those possessed by the subjects in this study are required for chimpanzees to copy human knapping and flake-use demonstrations *and/or* to enable them to innovate these behaviours. However, another possibility is that the chimpanzees in this study may not have had enough time to develop the behaviour, which perhaps may have emerged instead in long-term tests^37^. Indeed, wild chimpanzees often take years to acquire some of their skills, such as nut-cracking^6,7^. However, given the baseline low motivation levels of the chimpanzees (see below), it is unlikely that even longer testing periods would yield different results (see also^38^, for an overview of how long innovations can be expected to take in apes). Overall, it is therefore possible that some of the chimpanzees were sufficiently enculturated, but may have required longer periods of demonstrations to develop these behaviours. Note that both the enculturated bonobos and the orangutan who previously showed both target behaviours^14–16^ received longer periods of training, and potentially of demonstrations as well (although the exact number of hours of demonstrations and/or training is unclear from the original papers).

As mentioned, three of our subjects have unclear rearing histories. Therefore, it is also conceivable that some (or all) may have been deprived, instead of being enculturated, by living in ‘unnatural conditions’. Deprivation can have the opposite effect of enculturation on cognition, hindering an individual’s ability to innovate novel behaviours^39^—and potentially also leading to reduced social learning abilities. This, too, could explain the negative findings of this study for most of the subjects.

Future studies could try to better assess the enculturation levels of subjects with unknown backgrounds by using recently proposed methods to experimentally investigate enculturation (e.g., tracking the chimpanzees’ attention and actions compared to those of models performing the behaviour^40^; although see below for a discussion on human versus conspecific models). Unfortunately, we did not have the resources to carry-out these additional tests for the current study. In general, running the types of studies such as the one described here are very time-intensive and logistically difficult (which might also explain why all previous studies had very small sample sizes). Therefore, often compromises must be made, such as including chimpanzees in the sample that have mixed rearing backgrounds.

It is also possible that there is a species effect for these behaviours, in which great apes other than chimpanzees are capable of flake manufacture and use. Indeed, orangutans seem able to develop two main precursor behavioural types (flake production and their use as tools^18^). As only four chimpanzees were tested here, it is also possible that our negative findings were due to small sample size. However, previous studies on apes had smaller sample sizes than ours, but were successful (see above). Nevertheless, ideally, future studies should test larger groups of chimpanzees, including (ethical considerations permitting) fully enculturated and unenculturated subjects, controlling for the factors outlined above. If the hypothesis that enculturation is a key factor is correct, then the former should produce target behaviours at much higher rates than the latter.

Another explanation for our negative findings could be carry-over effects from the baseline test^19^ that we ran with these subjects prior to the current study. Namely, if present, these order effects may have resulted in the chimpanzees losing sufficient motivation for the task to benefit from the demonstrations provided here. To test for this possibility, unenculturated naive chimpanzees should be tested elsewhere again, starting directly with social learning conditions—perhaps even immediately with full demonstrations of the target behaviours. The chimpanzees in this study seemed to show a numerically low baseline level of motivation to interact with the provided materials (however there was no statistically significant decline between the interaction rates in our previous study^19^ and the conditions implemented in the current one). Although these chimpanzees receive regular enrichment tasks prepared by volunteers working at the sanctuary, they have not participated in research tasks like the one used in this study. Therefore, these particular subjects are relatively unfamiliar with puzzle boxes. This lack of experience may be an explanation for the chimpanzees’ low motivation to spend extended periods manipulating the testing materials we provided them. It could also be that the chimpanzees were not motivated to make cutting tools because they were not dependent on the food inside the box, as they receive a regular and varied diet from their keepers. However, according to the “the captivity effect” hypothesis, captive animals are more likely to innovate behaviours compared to their wild counterparts^41^, rendering this explanation unlikely. This account also does not explain why chimpanzees engage in other tasks in similar situations^38,42^ or why other captive ape studies—none of which deprived the apes of food—reported the development of the target abilities.

Despite insufficient levels of motivation being a potential explanation, the subjects did spend time manipulating the puzzle box and the materials. The subjects opened the puzzle box and consumed the food inside it when they could do so without tools (e.g., in the loose rope condition). Therefore, the chimpanzees clearly had some motivation to engage in the task^38^. Indeed, various types of manipulation were observed over the course of the study. This suggests that whilst the task was perhaps too opaque (at least for the subjects tested here) and may have limited the chimpanzees’ motivation to interact with it for extended periods, it was not ignored by the subjects. For example, one of the chimpanzees attempted to use a core on the rope in one of the earlier social learning conditions, suggesting that they had understood that the rope was an important component of the box, and perhaps even that it had to be removed to retrieve the reward. Note that the apparatus used in this study was modelled on the ones successfully used in previous similar studies^14–16^. However, future studies could provide testing apparatuses designed and tested to be even less complex and/or opaque to examine whether a simpler, or more intuitive, set-up encourages further manipulation of the materials (thus hopefully increasing the likelihood of innovation of the target behaviour).

Some of the methods to make flakes would have required the chimpanzees to manipulate and coordinate two tools at the same time (stone core and hammerstones) using one or both hands. Bimanual manipulation like this has been found to be rare in chimpanzees, indeed most of their natural behaviours require the use of only one hand at a time^43^ (although see also^44,45^). In a study on the manipulative abilities of captive chimpanzees and bonobos, it was reported that chimpanzees preferred to use only one hand, even when manipulating more than one object^46^. The captive bonobos tested in the same study instead showed a higher propensity towards bimanual activities, perhaps due to the fact that bonobos travel more arboreally than chimpanzees in the wild, therefore requiring the use of both hands^46^. In this regard, the difference in preference for unimanual and bimanual activities between species may provide yet another alternative explanation for the bonobos’ success in earlier studies and the failure of the chimpanzees in the current study. However, bimanual techniques were not the only method available to the chimpanzees to make flakes, for example, they could have used the dropping/throwing technique used by Kanzi^15^ and some modern humans^47^ (and potentially early hominins too^48^) which can be carried-out with one or two hands.

Another explanation for the negative results of this study could be that the chimpanzees were all too old, or more generally out of a potential sensitive learning periods, for the development of this behaviour (e.g., see^49^). Although the experience of the chimpanzees we tested in relation to stone tools is unknown (as we cannot fully account for some of the chimpanzees’ experience before arriving at Chimfunshi), it is likely that they were not familiar with stones as tools in general. Previous studies have found that exposure to stones as tools during a specific sensitive learning period has a positive effect on the development of stone tool-use behaviour later on in life across primates^49,50^. Furthermore, the development of bimanual manipulation (though see also above) has been observed to take several years of practice (and/or development) in chimpanzees (e.g.,^51^. One of the few behaviours that often involves the use of two hands and multiple objects in the wild is nut-cracking^52^. When studying the development of this behaviour in juvenile chimpanzees, Inoue-Nakamura & Matsuzawa^51^ found that juvenile chimpanzees were initially unable to carry out the bimanual manipulations required to crack nuts, or to correctly implement the various steps needed for the behaviour. It takes approximately 3-5 years of development and/or practice with the objects for wild chimpanzees to acquire these manipulative abilities^51^. Therefore, if the chimpanzees in this study were not exposed to stones during sensitive stages of their development, they may not have been able to recognize the provided stones as potential tools even after demonstrations of their properties, or to develop the bimanual manipulation abilities required for some of the knapping techniques (although, as mentioned above, only one hand is required for some techniques, such as Kanzi’s throwing/dropping technique^15^).

Lastly, the chimpanzees may not have recognized the human demonstrator as a salient enough model to copy but might have attended to a conspecific model showing the target behaviour instead (if such a model had been implemented). Data from an eye-tracking study with chimpanzees and human demonstrators found that chimpanzees paid more attention to conspecific social cues than to human ones, suggesting that chimpanzees may extract more referential information from conspecifics than from humans^53^. However, these studies do not suggest that chimpanzees will not attend human models at all (indeed, in other tasks, humans proved to be valid models; e.g.,^54^ and the previously tested apes learnt from humans, although this may have also been due to their enculturated status). As none of the chimpanzees showed any behaviours even approximating the target abilities, we could not explore this possibility by providing a conspecific model, but this would be an interesting avenue for future studies, perhaps using clicker training (and/or, where possible, moulding) to prepare demonstrators (see however^55^ in which chimpanzees failed to copy conspecific behaviours).

In conclusion, all the above are alternative explanations for the negative findings of this study. This does not mean, however, that all of these are equally likely, or mutually exclusive. Given the available data from flake production and use studies in apes, it is likely that alongside some of the explanations mentioned above, it is possible that as in the case of previously tested bonobos^15,16^ and one orangutan^14^, human training *and/or* demonstrations are required for the full flake manufacture and use behaviour to emerge. In addition however, there must also be a sufficiently high level of human enculturation before great apes are able to develop this behaviour *or* to copy these from humans. Indeed, these higher levels of enculturation are known to increase both the social and the individual learning abilities of affected apes, equipping them with the ability to acquire behaviours outside of the realm of possibilities of their unenculturated conspecifics.

## Data Availability

All the data collected for this study can be found on the Open Science Framework platform, following this link: https://osf.io/e24hk/?view_only=2d2b5ca6af2d41fd92c1da8d8e1a410c.
